# Investigating the association between neuroticism and adverse obstetric and neonatal outcomes

**DOI:** 10.1038/s41598-019-51861-y

**Published:** 2019-10-29

**Authors:** Cathrine Axfors, Patricia Eckerdal, Helena Volgsten, Anna-Karin Wikström, Lisa Ekselius, Mia Ramklint, Inger Sundström Poromaa, Alkistis Skalkidou

**Affiliations:** 1 Department of Neuroscience, Psychiatry, Uppsala University, Uppsala, Sweden; 2 Department for Women’s and Children’s Health, Uppsala University, Uppsala, Sweden

**Keywords:** Medical research, Epidemiology

## Abstract

Neuroticism is not only associated with affective disorders but also with certain somatic health problems. However, studies assessing whether neuroticism is associated with adverse obstetric or neonatal outcomes are scarce. This observational study comprises first-time mothers (n = 1969) with singleton pregnancies from several cohorts based in Uppsala, Sweden. To assess neuroticism-related personality, the Swedish universities Scales of Personality was used. Swedish national health registers were used to extract outcomes and confounders. In logistic regression models, odds ratios (ORs) with 95% confidence intervals (CIs) were calculated for the outcomes by an increase of 63 units of neuroticism (equalling the interquartile range). Analyses were adjusted for maternal age, educational level, height, body mass index, year of delivery, smoking during pregnancy, involuntary childlessness, and psychiatric morbidity. Main outcomes were mode of delivery, gestational diabetes mellitus, gestational hypertension, preeclampsia, induction of delivery, prolonged delivery, severe lacerations, placental retention, postpartum haemorrhage, premature birth, infant born small or large for gestational age, and Apgar score. Neuroticism was not independently associated with adverse obstetric or neonatal outcomes besides gestational diabetes. For future studies, models examining sub-components of neuroticism or pregnancy-specific anxiety are encouraged.

## Introduction

One of the most studied personality domains is neuroticism, and high levels are more common in women than in men^1^. Individuals with high scores of neuroticism typically describe themselves as anxious, vulnerable to stress, lacking self-confidence, and easily frustrated^2^. Neuroticism is associated with negative wellbeing^3^ and greater perceived need for health care^4^. Furthermore, neuroticism predisposes for major depression and anxiety disorders^5–8^. Studies report that individuals with high neuroticism experience more stressful life events than those with low neuroticism^9^ and are more sensitive to the depressogenic effects of adversity^10^. In addition to the well-studied relationship with mental disorders, neuroticism has been associated with certain somatic health problems and mortality^11–17^. Patients high in neuroticism more often express somatic complaints without medical explanation^18,19^ and report lower quality of life^20^.

During pregnancy and after childbirth, women with high neuroticism report higher pregnancy-related anxiety^21^, fear of vaginal delivery^22^, a more negative birth experience^23^, and a larger number of depressive symptoms^24^. Yet, studies exploring the role of neuroticism in obstetric and neonatal complications are limited. A few reports suggest that high neuroticism or corresponding measures are associated with negative outcomes such as preterm delivery contractions^25^, preterm birth^26^, foetal growth restriction^27^, and foetal distress^22,23^. One study found associations between neuroticism and epidural analgesia, prolonged delivery, severe birth canal tears, assisted vaginal delivery, and emergency caesarean section^23^. Nevertheless, results are inconsistent, with some investigations reporting no associations with preterm birth or low birthweight^28,29^.

Potential mediators may be considered for the association between neuroticism and obstetric and neonatal complications. First, high levels of stress during delivery are associated with prolonged delivery^30^, which may predispose for instrumental vaginal delivery, emergency caesarean section, postpartum haemorrhage, and low neonatal Apgar scores, a method to quickly summarize the health of a newborn^31^. Second, pregnant women reporting high neuroticism less often quit smoking^32^, and smoking increases the risk of several obstetric and neonatal complications (e.g., preterm delivery and low birthweight)^33^. Third, while obesity and the metabolic syndrome are overrepresented in individuals with high neuroticism^16,34^, maternal obesity conveys a heightened risk of gestational diabetes mellitus (GDM), preeclampsia, instrumental vaginal delivery, caesarean section, and certain foetal outcomes (e.g., large for gestational age (LGA), foetal distress, and intrauterine foetal death (IUFD))^35^. Furthermore, cardiovascular disease is associated with neuroticism^15,17^, possibly extending to placenta-related disorders (e.g., gestational hypertension, preeclampsia, and placental abruption), and foetal outcomes (e.g., spontaneous premature birth and small for gestational age (SGA)). Lastly, antenatal depression, which is associated with adverse neonatal outcomes, e.g., GDM, caesarean section, preeclampsia, premature birth, and SGA^36–38^, might also act as a mediator.

The present study aimed to assess whether neuroticism is associated with adverse obstetric or neonatal outcomes.

## Results

### Descriptive statistics

Delivery started spontaneously for 77.7% of the women and ended with non-instrumental vaginal delivery for 71.3%. Other descriptive statistics are presented in Table 1.

**Table 1:** Participants descriptive information (n = 1969).

Variable	N (%), or mean [SD] mdn	Missing
Neuroticism score	294.9 [45.1] 292.0	0
Maternal age (year)		0
14–24	633 (32.1)	
25–30	712 (36.2)	
31–43	624 (31.7)	
No college or university education	713 (36.3)	3
Maternal height (cm)		109
143–164	649 (34.9)	
165–169	577 (31.0)	
170–186	634 (34.1)	
BMI at first antenatal care visit (kg/m^2^)		244
<18.5	42 (2.4)	
18.5–25.0	1168 (67.7)	
>25.0	515 (29.9)	
Year of delivery		0
1984–1996	156 (7.9)	
1997–2012	1813 (92.1)	
Smoking at first antenatal care visit or at gestational week 32	169 (9.0)	81
Chronic somatic disease^a^	27 (1.4)	0
Involuntary childlessness^b^	318 (16.2)	0
Psychiatric morbidity^c^	176 (8.9)	0
Vaginal delivery, non-instrumental	1404 (71.3)	0
Vaginal delivery, vacuum extraction	261 (13.3)	0
Any caesarean section	304 (15.4)	0
Elective caesarean section	80 (4.1)	0
Emergency caesarean section	224 (11.4)	0
Deliveries starting with emergency caesarean section	25 (1.2)	0
Gestational diabetes mellitus	10 (0.5)	0
Gestational hypertension or Preeclampsia	115 (5.8)	0
Induction of delivery	334 (17.0)	0
Prolonged delivery^d^	444 (23.8)	0
Severe tears^e^	134 (8.0)	0
Placental retention^e^	56 (3.4)	0
Postpartum haemorrhage	124 (6.3)	0
Premature birth <37 weeks^f^	99 (5.0)	0
Small for gestational age <10^th^ percentile^f^	116 (5.9)	8
Large for gestational age >90^th^ percentile^f^	139 (7.1)	8
Apgar 5 minutes <7	23 (1.2)	0
Composite worst-case variable^g^	47 (2.4)	0

Note. Abbreviations: body mass index (BMI), median (mdn), standard deviation (SD).

^a^Pre-gestational hypertension, diabetes mellitus or chronic kidney disease. ^b^*In vitro* fertilization or self-reported involuntary childlessness >1 year. ^c^Mental disorders due to psychoactive substance use’, ‘affective disorders’, ‘anxiety, stress-related and somatoform disorders’, ‘eating disorders’, ‘personality disorders’, ‘disturbances of activity and attention’, or prescription of antidepressant or anxiolytic drugs during pregnancy. ^d^Excluding deliveries starting with emergency caesarean section (n = 25) and elective caesarean section. ^e^Excluding any caesarean section. ^f^Excluding stillborn (n = 3). ^g^Stillborn, eclampsia, severe preeclampsia, premature birth <32 weeks, small for gestational age below minus 2.5 standard deviations (0.6%), placental abruption.

### Main results

Young age, lower educational level, underweight, overweight, smoking during pregnancy, and psychiatric morbidity were crudely associated with neuroticism (Table 2). In the logistic regression models (Table 3) a significant crude association was found between neuroticism and GDM. Women with higher neuroticism scores (by 63 units, corresponding to the IQR) had 2.5 times higher odds of GDM. After adjustment for maternal characteristics, the OR was estimated at 3.6. After considering even psychiatric morbidity, the association was not significant in the unimputed dataset; however, it was statistically significant in the dataset with imputed missing data (see below). In addition, crude associations were present with vacuum extraction and placental retention but not after adjustment for maternal characteristics. No associations with neuroticism were found on the other study outcomes.

**Table 2:** Potential confounder variables and bivariable associations with neuroticism.

Variable	Neuroticism mdn (IQR)	*p*
*Age (years)*		<0.001
14–24	309 (68)	
25–30	285 (60)	
31–34	287 (60)	
*Educational level*		<0.001
College or university	283 (58)	
No college or university	305 (66)	
*Maternal height (cm)*		Ns
143–164	295 (61)	
165–169	292 (61)	
170–186	288 (64)	
*BMI at first antenatal care visit (kg/m* ^2^ *)*		<0.001
<18.5	311 (51)	
18.5–25.0	289 (59)	
>25.0	297 (68)	
*Year of delivery*		Ns
1984–1996	287 (58)	
1997–2012	292 (63)	
*Smoking at first antenatal care visit or at gestational week 32*		<0.001
No	290 (61)	
Yes	308 (82)	
*Chronic somatic disease* ^*a*^		Ns
No	292 (63)	
Yes	319 (66)	
*Involuntary childlessness* ^*b*^		Ns
No	291 (63)	
Yes	294 (61)	
*Psychiatric morbidity* ^*c*^		<0.001
No	289 (62)	
Yes	318 (68)	

Note. Abbreviations: body mass index (BMI), interquartile range (IQR), median (mdn), non-significant (ns). Mann-Whitney *U* test for binary variables; Kruskal-Wallis test for variables with three categories.

^a^Pre-gestational hypertension, diabetes mellitus or chronic kidney disease. ^b^*In vitro* fertilization or self-reported involuntary childlessness >1 year. ^c^Mental disorders due to psychoactive substance use’, ‘affective disorders’, ‘anxiety, stress-related and somatoform disorders’, ‘eating disorders’, ‘personality disorders’, ‘disturbances of activity and attention’, or prescription of antidepressant or anxiolytic drugs during pregnancy.

**Table 3:** Logistic regression-derived odds ratios (ORs) with 95% confidence intervals (CIs) for obstetric and neonatal outcomes by an increase of 63 units of neuroticism (equaling the interquartile range).

Outcome	Cases	Total	Crude OR (95% CI)	Adj OR (95% CI)	Adj OR (95% CI)
				Model 1	Model 2
Vaginal delivery, non-instrumental	1404	1969	1.09 (0.95–1.26)	1.08 (0.92–1.28)	1.07 (0.91–1.26)
Vaginal delivery, vacuum extraction	261	1969	0.79 (0.65–0.95)	0.82 (0.66–1.01)	0.85 (0.68–1.05)
Any caesarean section	304	1969	1.06 (0.89–1.26)	1.06 (0.87–1.30)	1.05 (0.85–1.28)
Elective caesarean section	80	1969	1.03 (0.76–1.41)	0.97 (0.68–1.39)	0.94 (0.65–1.34)
Emergency caesarean section	224	1969	1.07 (0.88–1.29)	1.09 (0.87–1.38)	1.09 (0.87–1.38)
Gestational diabetes mellitus	10	1969	2.53 (1.20–5.33)	3.57 (1.32–9.65)	2.70 (0.92–7.95)^d^
Gestational hypertension or Preeclampsia	115	1969	1.07 (0.82–1.39)	1.13 (0.85–1.50)	1.12 (0.84–1.50)
Induction of delivery	334	1969	1.04 (0.88–1.23)	1.06 (0.88–1.28)	1.01 (0.84–1.22)
Prolonged delivery	444	1969	0.95 (0.82–1.11)	0.99 (0.84–1.17)	0.99 (0.83–1.17)
Severe tears	134	1969	0.91 (0.71–1.17)	0.88 (0.67–1.17)	0.92 (0.70–1.22)
Placental retention	56	1969	0.66 (0.44–0.99)	0.70 (0.45–1.09)	0.71 (0.46–1.11)
Postpartum haemorrhage	124	1969	0.95 (0.74–1.23)	0.97 (0.73–1.29)	0.94 (0.70–1.26)
Premature birth <37 weeks^a^	99	1966	0.87 (0.65–1.16)	0.95 (0.69–1.33)	0.96 (0.69–1.35)
Small for gestational age <10^th^ percentile^a,b^	116	1958	1.12 (0.87–1.45)	1.15 (0.86–1.54)	1.11 (0.82–1.49)
Large for gestational age >90^th^ percentile^a,b^	139	1958	1.14 (0.90–1.45)	1.07 (0.82–1.41)	1.06 (0.80–1.39)
Apgar 5 minutes <7	23	1969	0.77 (0.42–1.41)	0.92 (0.48–1.77)	0.87 (0.45–1.70)
Composite worst-case variable^c^	47	1969	0.86 (0.57–1.30)	0.86 (0.54–1.38)	0.83 (0.51–1.34)

Note. Model 1 adjusted (adj) for maternal age at childbirth, educational level, height, body mass index at first health care visit during pregnancy, year of delivery, smoking at first antenatal care visit and/or at gestational week 32. Model 2 also adjusted for psychiatric morbidity. ^a^Excluding stillborn (n = 3). ^b^According to Swedish sex-specific reference curves. ^c^Stillborn, eclampsia, severe preeclampsia, premature birth < 32 weeks, Small for gestational age below −2.5 SD (0.6%), placental abruption. ^d^The association was significant in the analyses with imputed missing values.

Results were equivalent after the exclusion of non-applicable cases for certain outcomes (Table S1). Imputation of missing values for BMI, maternal height, and smoking during pregnancy yielded similar results as the non-imputed models, except that the association between neuroticism and GDM remained significant after adjustment for psychiatric morbidity.

## Discussion

Despite research linking neuroticism with diverse negative health conditions, our study in general found no associations between neuroticism and a range of obstetric or neonatal outcomes in first-time mothers with singleton pregnancies, besides a positive association between neuroticism and GDM.

Thus far, this is the largest study on neuroticism and obstetric and neonatal complications. Yet, a limitation of the study pertains to the fact that some outcomes, including GDM, are rare and the number of observations is often very small; these would preferably be examined in even larger settings or using another study design. We used a combination of self-report personality measures and national register data on complication rates, which avoids the potential mono-method and recall bias that could occur when complications are self-reported. Nonetheless, some diagnoses may be falsely reported.

Although the gathering of personality data across different study populations and settings possibly led to a varied sample, with women of different ages and life situations, and even though the personality scores do not seem to deviate from previously reported Swedish norms^39^, we cannot ascertain their representability of pregnant women at the time.

Even though the applied instrument for personality holds a moderate concordance with the Big Five counterpart^40^, there may be differences. The SSP-neuroticism includes the aspects of “lack of assertiveness”, “embitterment”, and “mistrust”, which might possibly be conceptualized as belonging to the personality domain (non-)agreeableness in the Big Five.

Furthermore, women born outside Sweden were underrepresented in that their first childbirth was more likely not to be recorded in Swedish national registers. We cannot exclude the possibility that associations between neuroticism and adverse obstetric or neonatal outcomes may be present among less socioeconomically advantaged women.

In this study maternal factors (younger age, lower education, underweight and overweight, smoking, and psychiatric morbidity) were associated with neuroticism in accordance with earlier studies^16,23,32,34,41^. However, for obstetric and neonatal outcomes, no robust associations were seen. Some of our results contradict a study by Johnston and colleagues^23^, which shows associations with self-reported emergency caesarean section, failure to progress, foetal distress, and episiotomy or severe birth canal tears. However, basing the complication rates on medical health registers, such as in our study, has an advantage over self-report rates because the latter may introduce recall bias. A German study demonstrates that only childbirth-specific anxiety, not general anxiety, is associated with longer delivery duration^42^. Although neuroticism is not equivalent to general anxiety, these concepts share the feature of non-specified distress. It has been argued that pregnancy-specific anxiety is an entity of its own and more strongly predicts adverse obstetric and neonatal outcomes^43^, a claim which is consistent with the present findings. In our study women with high neuroticism did not give birth more often via caesarean section (neither by elective nor by emergency caesarean section). Yet, in line with national recommendations promoting a vaginal delivery for pregnant women without specific maternal or foetal indications, the caesarean section rates are lower in Sweden compared with other European countries^44^. Possibly, caesarean section on maternal request in women with high neuroticism might be more common in countries with higher rates of caesarean section.

Neither preterm birth nor SGA infant birth were more common in women with high neuroticism. Whereas the associations between antenatal depression and preterm birth and SGA are well-established^36^, results are mixed regarding associations with maternal anxiety. In a large population-based Norwegian cohort study Vollrath *et al*.^26^ report substantial associations between trait anxiety and late preterm birth. Unlike the present results, no adjustment was made for psychiatric morbidity. Nevertheless, not even in our crude analysis was there any relation between neuroticism and preterm birth. Notably, their four-item trait anxiety measure was used exclusively during pregnancy^26^. In the current study, neuroticism report dates were removed to secure the anonymity of the participants. Therefore, we could not ascertain whether neuroticism was measured before, during, or after pregnancy. On the one hand, personality is relatively consistent in adulthood^45^, with a tendency of normative change^46^. However, studies exploring the course of trait anxiety across the peripartum period are few and contradictory, some reporting no differences^47,48^ and one reporting increasing levels^49^. Our null findings on preterm birth are supported by two studies^27,29^ that measure anxiety-related personality traits during pregnancy; however, one of these^27^ notes a relationship with low birthweight. Perhaps a state-dependent component of maternal distress, which might better be detected by other measure than personality, may carry more valence for preterm birth and low birthweight. Using psychologists’ assessment of distress during pregnancy, Rondó *et al*.^50^ find associations with premature birth as well as with low birthweight.

To our knowledge, an association between neuroticism and GDM has not been previously reported. However, in a recent meta-analysis, an association of GDM and postpartum depression is reported^37^, and in the Norwegian cohort study on preterm birth^26^, a crude association between trait anxiety and GDM is incidentally mentioned. Still, the relation presented in our article was based on a small number of cases (n = 10) and should thus be regarded as preliminary. As a speculation, a possible pathway between neuroticism and GDM might be heightened stress levels and chronic inflammation that induces insulin resistance^51^. Moreover, in our cohort, as well as in others^16,34^, there was a positive correlation between neuroticism and obesity. The latter, together with other maternal characteristics and earlier psychiatric morbidity, could act as mediators in the association between neuroticism and GDM.

The prevalence of GDM was slightly lower in our cohort (0.5%) as compared with previous Swedish figures^52^. Some maternal diagnoses may be under-reported in the Medical Birth Register (MBR)^53^ from which the data were drawn. Nonetheless, the discrepancy could also be explained by the sample constitution as a low-risk cohort for GDM taking into account educational level and country of birth^52^.

Future studies could acknowledge a greater complexity of personality than given by the one domain of neuroticism. On the domain level, there was no association with obstetric or neonatal complications. Studies using sub-components of neuroticism or the combination of neuroticism and pregnancy-specific anxiety measures might advance this field of inquiry still further.

## Conclusion

Neuroticism, while associated with age, education, body mass index (BMI), and psychiatric history, was not an independent risk factor for adverse obstetric or neonatal outcomes, besides an increased risk of GDM. Future studies are needed to replicate this finding and they may choose to even investigate neuroticism sub-components or both neuroticism and pregnancy-specific anxiety.

## Methods

### Sample

In the years 2005–2011 personality measures were distributed as part of several projects based in Uppsala, Sweden, with female participants. The projects investigated oral contraceptive use (n = 118)^54^, infertility (n = 320)^55^, induced abortion (n = 1320)^56^, premenstrual mood disorder (n = 44)^57^, and wellbeing during pregnancy (n = 1017)^24^. More than two-thirds of these women were residing in Uppsala at the time of data collection while the remaining third were recruited from Obstetrics and Gynaecology centres in Umeå, Örebro, Linköping, and Stockholm, or referred to Uppsala from nearby counties.

**Figure 1:**
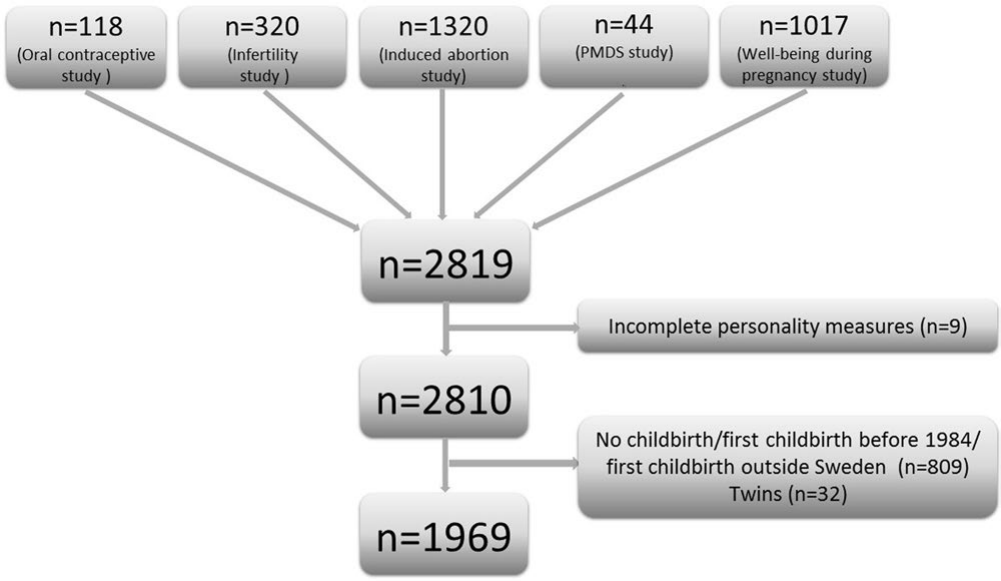
Flowchart of the included studies and participants.

Of the original cohort of 2819 women, the Swedish personal identity numbers^58^ of 2810 with full data on personality measures were used to link information from several Swedish health registers: the Medical Birth Register (MBR)^53^ from 1984–2012 concerning participants’ first childbirth (previous somatic and psychiatric health, medication at first antenatal booking, information on pregnancy and delivery), the Patient Register^59^ from 5 years before childbirth to 1 year after childbirth (diagnoses from hospital admissions and outpatient clinic visits), the Swedish government agency Statistics Sweden (socioeconomic factors), and the Prescribed Drug Register^60^ (prescribed antidepressants and anxiolytics during pregnancy). Women who had not given birth or whose first childbirth took place before 1984 or outside Sweden (n = 809) and twin pregnancies (n = 32) were excluded, leaving a final sample of 1969 participants (Fig. 1). Most of the participants had their first childbirth in the later part of the study period (mean year = 2006, SD = 5.3 years, median year = 2008).

### Measures and study variables

Neuroticism was self-reported in the Swedish universities Scales of Personality (SSP)^39^, a revised version of the Karolinska Scales of Personality, with a reduced number of items and improved psychometric quality. The SSP has a similar factor structure in pregnant women as in the reference population^24^, including three personality domains: neuroticism, aggressiveness, and sensation seeking (the latter two were not addressed in this study). The neuroticism domain of the SSP contains 42 statements rated on four-point scale from “not applicable at all” to “applies completely”. The items form six scales named after their content: Somatic Trait Anxiety, Psychic Trait Anxiety, Stress Susceptibility, Lack of Assertiveness, Embitterment, and Mistrust. The SSP scores on each scale were transformed into normative T-scores, adjusted for age and gender, based on a representative Swedish non-patient sample^61^. The neuroticism score equals the sum of the T-scores from the six scales constituting this domain (median = 292, IQR = 63, range = 194–474). Internal consistency (Cronbach’s α) of the six subscales range from 0.74–0.82^39^. In the present material, the whole 42-statement neuroticism domain had α = 0.94.

Obstetric and neonatal outcomes were obtained from the MBR, a register that is based on health records from maternal health services and obstetrics and gynaecology centres. Diagnostic codes from International Statistical Classification of Diseases and Related Health Problems (ICD)^62^ versions 8–10 were used to categorize the outcomes (Table S2). Additional to diagnostic codes, MBR-specific variables relating to first visit during pregnancy and delivery were also used. Obstetric outcomes included non-instrumental vaginal delivery, vaginal delivery assisted by vacuum extraction (VE; no case of forceps use was reported), any caesarean section, elective caesarean section, emergency caesarean section, GDM, gestational hypertension, preeclampsia, induction of delivery, prolonged delivery, severe tears (tear of the anal sphincter, cervix rupture, or complex vaginal lacerations), placental retention, and postpartum haemorrhage. Neonatal outcomes comprised premature birth (before gestational week 37 + 0), SGA (below the 10^th^ percentile, according to the Swedish sex-specific reference curves for gestational age^63^), LGA (above the 90^th^ percentile), and a 5-minute Apgar score below seven. Finally, a composite worst-case variable was created out of IUFD, eclampsia, severe preeclampsia, premature birth before gestational week 32 + 0, SGA < −2.5 SD (lowest 0.6%), and placental abruption. Gestational hypertension and preeclampsia were grouped together because of their low frequency and common aetiology.

Information about possible confounders was extracted from Statistics Sweden, the MBR, the Patient Register, and the Prescribed Drug Register. These variables included maternal age at childbirth, educational level, maternal height, BMI at first antenatal care visit, year of delivery, smoking reported at the first antenatal care visit or in gestational week 32, chronic somatic disease (pre-gestational hypertension, diabetes mellitus, or chronic kidney disease), involuntary childlessness (≥1 year or use of *in vitro* fertilization), and psychiatric morbidity. Psychiatric morbidity consisted of ICD codes^62^ from hospital admissions and out-patient clinics from 5 years before the delivery to 1 year after delivery for the following: ‘mental disorders due to psychoactive substance use’, ‘affective disorders’, ‘anxiety, stress-related and somatoform disorders’, ‘eating disorders’, ‘personality disorders’, ‘disturbances of activity and attention’, and the prescription of antidepressant or anxiolytic drugs during pregnancy. Maternal age and height were categorized into approximately three equal groups. BMI was classified according to the World Health Organization criteria (underweight <18.5 kg/m^2^, normal range 18.5–25 kg/m^2^, overweight >25 kg/m^2^).

### Statistical analysis

Logistic regression models were used to explore the association between neuroticism as a continuous exposure and each of the obstetric and neonatal outcomes, which were all binary (two-categorical, coded as 0 or 1). Odds ratios (ORs) for the outcomes with 95% confidence intervals (CIs) were calculated for a 63-unit increase in neuroticism, which equals the interquartile range (IQR). Because the model is linear, this may also be interpreted as the OR comparing women at the 75^th^ and 25^th^ percentiles of neuroticism. Crude and adjusted models were estimated. All variables listed above as possible confounders were applied for adjustment, except for chronic somatic disease because of its low frequency. Since psychiatric disorders may be considered as a potential mediator between personality and outcome, we chose to add the variable psychiatric morbidity only in a second adjusted model. In Table 2, bivariable associations between the possible confounders and neuroticism are exemplified (Mann-Whitney *U* for binary variables, Kruskal-Wallis test for three-categorical variables). In a separate version of the models, missing data were replaced by multiple imputation (regarding the variables with more than a few missing items: BMI, maternal height, and smoking during pregnancy). We did not perform any correction for multiple tests, as we considered the outcomes as distinct and separate, most without bearing on each other.

Additional analyses were made for some of the outcomes, where “non-applicable” cases were excluded in order to test the robustness of the results. In contrast with the main analyses, where the denominator was “all deliveries”, the exclusion of non-applicable cases changed the denominator to “vaginal deliveries” for the outcomes VE, severe tears and placental retention (excluded women undergoing elective or emergency caesarean section, as they could not be at risk for e.g. vaginal tears). Regarding emergency caesarean section, the denominator was changed to “deliveries intended as vaginal” (excluded elective caesarean section). Similarly, for induction of delivery and for prolonged delivery, the denominator was changed to “deliveries intended as vaginal” (excluded elective caesarean section as well as those deliveries starting with emergency caesarean section). For elective caesarean section, deliveries starting with emergency caesarean section were excluded since they may have been intended as elective caesarean sections.

### Details of ethics approval

All women who gave their written informed consent were told about the course and aim of the individual original studies. The investigation was carried out in accordance with the Declaration of Helsinki and the study protocol was approved by the Regional Research and Ethics Committee of Uppsala (Dnr 2014/092) in June 2014.

## Supplementary information

Supplementary tables

## References

[1] Kendler KS, Gatz M, Gardner CO, Pedersen NL (2006). Personality and major depression: a Swedish longitudinal, population-based twin study. Arch Gen Psychiatry.

[2] Caspi A, Roberts BW, Shiner RL (2005). Personality development: stability and change. Annu Rev Psychol..

[3] Steel P, Schmidt J, Shultz J (2008). Refining the relationship between personality and subjective well-being. Psychol Bull.

[4] Seekles WM (2012). Personality and perceived need for mental health care among primary care patients. J Affect Disord.

[5] Bienvenu OJ, Hettema JM, Neale MC, Prescott CA, Kendler KS (2007). Low extraversion and high neuroticism as indices of genetic and environmental risk for social phobia, agoraphobia, and animal phobia. Am J Psychiatry.

[6] Bienvenu OJ (2001). Phobic, panic, and major depressive disorders and the five-factor model of personality. J Nerv Ment Dis.

[7] Hettema JM, Prescott CA, Kendler KS (2004). Genetic and environmental sources of covariation between generalized anxiety disorder and neuroticism. Am J Psychiatry.

[8] Rector NA, Richter MA, Bagby RM (2005). The impact of personality on symptom expression in obsessive-compulsive disorder. J Nerv Ment Dis.

[9] Boals A, Southard-Dobbs S, Blumenthal H (2015). Adverse events in emerging adulthood are associated with increases in neuroticism. J Pers.

[10] Kendler KS, Kuhn J, Prescott CA (2004). The interrelationship of neuroticism, sex, and stressful life events in the prediction of episodes of major depression. The American journal of psychiatry.

[11] Breslau N, Chilcoat HD, Andreski P (1996). Further evidence on the link between migraine and neuroticism. Neurology.

[12] Talley NJ, Boyce PM, Jones M (1998). Is the association between irritable bowel syndrome and abuse explained by neuroticism? A population based study. Gut.

[13] Muth ER, Koch KL, Stern RM (2000). Significance of autonomic nervous system activity in functional dyspepsia. Dig Dis Sci.

[14] Karaiskos, D. *et al*. Psychopathological and personality features in primary Sjogren’s syndrome–associations with autoantibodies to neuropeptides. *Rheumatology (Oxford*) **49**, 1762–1769, doi:10.1093/rheumatology/keq158 (2010). 10.1093/rheumatology/keq158 20525741

[15] Zotti AM, Bettinardi O, Soffiantino F, Tavazzi L, Steptoe A (1991). Psychophysiological stress testing in postinfarction patients. Psychological correlates of cardiovascular arousal and abnormal cardiac responses. Circulation.

[16] Phillips AC (2010). Neuroticism, cognitive ability, and the metabolic syndrome: The Vietnam Experience Study. J Psychosom Res.

[17] Shipley BA, Weiss A, Der G, Taylor MD, Deary IJ (2007). Neuroticism, extraversion, and mortality in the UK Health and Lifestyle Survey: a 21-year prospective cohort study. Psychosom Med.

[18] Costa PT, McCrae RR (1987). Neuroticism, somatic complaints, and disease: is the bark worse than the bite?. J Pers.

[19] Noyes R (2005). Relationship between hypochondriacal concerns and personality dimensions and traits in a military population. J Nerv Ment Dis.

[20] Chapman B, Duberstein P, Lyness JM (2007). Personality traits, education, and health-related quality of life among older adult primary care patients. J Gerontol B Psychol Sci Soc Sci.

[21] Peñacoba-Puente C, Carmona Monge FJ, Carretero Abellán I, Marín Morales D (2011). Effects of Personality on Psychiatric and Somatic Symptoms in Pregnant Women. Psychol Women Q.

[22] Handelzalts JE (2015). Personality, fear of childbirth and birth outcomes in nulliparous women. Arch Gynecol Obstet.

[23] Johnston RG, Brown AE (2013). Maternal trait personality and childbirth: the role of extraversion and neuroticism. Midwifery.

[24] Iliadis SI (2015). Personality and risk for postpartum depressive symptoms. Arch Womens Ment Health.

[25] Handelzalts JE (2016). Personality, preterm labor contractions, and psychological consequences. Arch Gynecol Obstet.

[26] Vollrath ME, Sengpiel V, Landolt MA, Jacobsson B, Latal B (2016). Is maternal trait anxiety a risk factor for late preterm and early term deliveries?. BMC Pregnancy and Childbirth.

[27] Chatzi L (2013). Maternal personality traits and risk of preterm birth and fetal growth restriction. European psychiatry: the journal of the Association of European Psychiatrists.

[28] Morisaki N, Fujiwara T, Horikawa R (2016). The Impact of Parental Personality on Birth Outcomes: A Prospective Cohort Study. PloS one.

[29] Schatz D (2000). The Relationship of Maternal Personality Characteristics to Birth Outcomes and Infant Development. Birth.

[30] Lederman RP, Lederman E, Work BA, McCann DS (1978). The relationship of maternal anxiety, plasma catecholamines, and plasma cortisol to progress in labor. Am J Obstet Gynecol.

[31] Kjaergaard H, Olsen J, Ottesen B, Dykes AK (2009). Incidence and outcomes of dystocia in the active phase of labor in term nulliparous women with spontaneous labor onset. Acta Obstet Gynecol Scand.

[32] Maxson PJ, Edwards SE, Ingram A, Miranda ML (2012). Psychosocial differences between smokers and non-smokers during pregnancy. Addictive behaviors.

[33] Andres RL, Day M-C (2000). Perinatal complications associated with maternal tobacco use. Seminars in Neonatology.

[34] Gerlach G, Herpertz S, Loeber S (2015). Personality traits and obesity: a systematic review. Obesity reviews: an official journal of the International Association for the Study of Obesity.

[35] Cedergren MI (2004). Maternal morbid obesity and the risk of adverse pregnancy outcome. Obstet Gynecol.

[36] Schaffir J (2018). Consequences of Antepartum Depression. Clin Obstet Gynecol.

[37] Arafa A, Dong JY (2019). Gestational diabetes and risk of postpartum depressive symptoms: A meta-analysis of cohort studies. J Affect Disord.

[38] Hinkle SN (2016). A longitudinal study of depression and gestational diabetes in pregnancy and the postpartum period. Diabetologia.

[39] Gustavsson JP (2000). Swedish universities Scales of Personality (SSP): construction, internal consistency and normative data. Acta Psychiatr Scand.

[40] Aluoja A (2009). Personality traits measured by the Swedish universities Scales of Personality: factor structure and position within the five-factor model in an Estonian sample. Nord J Psychiatry.

[41] Terracciano A, Costa PT (2004). Smoking and the Five-Factor Model of personality. Addiction.

[42] Reck C (2013). The influence of general anxiety and childbirth-specific anxiety on birth outcome. Arch Womens Ment Health.

[43] Bayrampour H (2016). Pregnancy-related anxiety: A concept analysis. International journal of nursing studies.

[44] Zeitlin, J. *et al*. *European Perinatal Health Report. The health and care of pregnant women and babies in Europe in 2010*. (Euro Peristat, 2013). 10.1136/jech.2009.087296 19679713

[45] Fraley RC, Roberts BW (2005). Patterns of continuity: a dynamic model for conceptualizing the stability of individual differences in psychological constructs across the life course. Psychol Rev.

[46] Roberts BW, Walton KE, Viechtbauer W (2006). Patterns of mean-level change in personality traits across the life course: a meta-analysis of longitudinal studies. Psychol Bull.

[47] Canals J, Esparó G, Fernández-Ballart JD (2002). How anxiety levels during pregnancy are linked to personality dimensions and sociodemographic factors. Personality and Individual Differences.

[48] Specht J, Egloff B, Schmukle SC (2011). Stability and change of personality across the life course: The impact of age and major life events on mean-level and rank-order stability of the Big Five. J Pers Soc Psychol.

[49] Jokela M, Kivimäki M, Elovainio M, Keltikangas-Järvinen L (2009). Personality and having children: A two-way relationship. J Pers Soc Psychol.

[50] Rondó PH (2003). Maternal psychological stress and distress as predictors of low birth weight, prematurity and intrauterine growth retardation. Eur J Clin Nutr.

[51] Joseph JJ, Golden SH (2017). Cortisol dysregulation: the bidirectional link between stress, depression, and type 2 diabetes mellitus. Annals of the New York Academy of Sciences.

[52] Lindqvist M, Persson M, Lindkvist M, Mogren I (2014). No consensus on gestational diabetes mellitus screening regimes in Sweden: pregnancy outcomes in relation to different screening regimes 2011 to 2012, a cross-sectional study. BMC Pregnancy Childbirth.

[53] National Board of Health and Welfare. The Swedish Medical Birth Register: a summary of content and quality. 2003-112-3. (2003).

[54] Borgstrom A, Odlind V, Ekselius L, Sundstrom-Poromaa I (2008). Adverse mood effects of combined oral contraceptives in relation to personality traits. European journal of obstetrics, gynecology, and reproductive biology.

[55] Volgsten H, Ekselius L, Poromaa IS, Svanberg AS (2010). Personality traits associated with depressive and anxiety disorders in infertile women and men undergoing *in vitro* fertilization treatment. Acta Obstet Gynecol Scand.

[56] Wallin Lundell I (2017). Neuroticism-related personality traits are associated with posttraumatic stress after abortion: findings from a Swedish multi-center cohort study. BMC women’s health.

[57] Gingnell M, Comasco E, Oreland L, Fredrikson M, Sundstrom-Poromaa I (2010). Neuroticism-related personality traits are related to symptom severity in patients with premenstrual dysphoric disorder and to the serotonin transporter gene-linked polymorphism 5-HTTPLPR. Arch Womens Ment Health.

[58] Ludvigsson JF, Otterblad-Olausson P, Pettersson BU, Ekbom A (2009). The Swedish personal identity number: possibilities and pitfalls in healthcare and medical research. European journal of epidemiology.

[59] National Board of Health and Welfare. Kvalitet och innehåll i patientregistret. Utskrivningar från slutenvården 1964–2007 och besök i specialiserad öppenvård (exklusive primärvårdsbesök) 1997–2007. (Quality and content of the Patient Register). 2009-125-15. (2009).

[60] Wettermark B (2007). The new Swedish Prescribed Drug Register–opportunities for pharmacoepidemiological research and experience from the first six months. Pharmacoepidemiology and drug safety.

[61] Willebrand M, Kildal M, Andersson G, Ekselius L (2002). Long-term assessment of personality after burn trauma in adults. J Nerv Ment Dis.

[62] World Health Organization. *Classification of Diseases (ICD)*, http://www.who.int/classifications/icd/en/ (2016).

[63] Marsal K (1996). Intrauterine growth curves based on ultrasonically estimated foetal weights. Acta paediatrica (Oslo, Norway: 1992).

