# Supplementary material for: Investigating the association between neuroticism and adverse obstetric and neonatal outcomes
Source: Sci Rep. 2019 Oct 29;9:15470. doi: 10.1038/s41598-019-51861-y (PMC6820798; doi:10.1038/s41598-019-51861-y)
Supplement: Supplementary file 1 — Supplementary tables [file 41598_2019_51861_MOESM1_ESM.docx]

Investigating the association between neuroticism and adverse obstetric and neonatal outcomes

**Supplementary tables**

Cathrine AXFORS_a_^1^, PhD., Patricia ECKERDAL_b_^1^, PhD., Helena VOLGSTEN_b_, Ph.D., Anna-Karin WIKSTRÖM_b_, Professor, Lisa EKSELIUS_a_, Professor, Mia RAMKLINT_a_, Associate Professor, Inger SUNDSTRÖM POROMAA_b_, Professor, Alkistis SKALKIDOU_b_, Professor

^1^ Contributed equally

^a^ Department of Neuroscience, Psychiatry, Uppsala University, Sweden

^b^ Department for Women’s and Children’s Health, Uppsala University, Sweden

Corresponding author: Patricia Eckerdal

Address: Department of Women’s and Children’s Health, Uppsala University. Akademiska Sjukhuset, SE-75185 Uppsala, Sweden

Work phone number: +46 18 611 5983

E-mail address: patricia.eckerdal@kbh.uu.se

**Table S1.** Additional models excluding non-applicable situations. Logistic regression-derived odds ratios (ORs) with 95% confidence intervals (CIs) for obstetric and neonatal outcomes by an increase of 63 units of neuroticism (equaling the interquartile range).

| **Outcome** | **Cases** | **Total** | **Crude OR (95% CI)** | **Adj OR (95% CI)** | **Adj OR (95% CI)** |
| --- | --- | --- | --- | --- | --- |
|  |  |  |  | **Model 1** | **Model 2** |
| **Vaginal delivery, vacuum extraction ^a,b^** | 261 | 1665 | **0.79 (0.65-0.96)** | 0.81 (0.65-1.01) | 0.83 (0.67-1.05) |
| **Elective cesarean section ^c^** | 80 | 1944 | 1.03 (0.76-1.41) | 0.97 (0.68-1.39) | 0.94 (0.65-1.35) |
| **Emergency cesarean section ^a^** | 224 | 1889 | 1.07 (0.88-1.30) | 1.10 (0.87-1.39) | 1.10 (0.87-1.38) |
| **Induction of delivery ^a,c^** | 334 | 1864 | 1.05 (0.89-1.23) | 1.07 (0.88-1.29) | 1.02 (0.84-1.23) |
| **Dystocia ^a,c^** | 444 | 1864 | 0.96 (0.82-1.11) | 0.99 (0.84-1.18) | 0.98 (0.83-1.17) |
| **Severe lacerations ^a,b^** | 134 | 1665 | 0.92 (0.72-1.18) | 0.88 (0.66-1.17) | 0.91 (0.69-1.22) |
| **Placental retention ^a,b^** | 56 | 1665 | 0.67 (0.45-1.00) | 0.68 (0.43-1.07) | 0.69 (0.44-1.08) |

*Note.* Model 1 adjusted (adj) for maternal age at childbirth, educational level, height, body mass index at first antenatal care visit, year of delivery, smoking at first antenatal care visit and/or at gestational week 32. Model 2 also adjusted for psychiatric morbidity. Values in boldface are significant at *p*<0.05.

^a^ Excluding elective cesarean section. ^b^ Excluding emergency cesarean section. ^c^ Excluding deliveries starting with emergency cesarean section (n=25).

**Table S2.** Origin of study variables from diagnostic coding systems.

| **Variable** | **ICD-9** | **ICD-10** | **KVÅ** | **Use of MBR data** |
| --- | --- | --- | --- | --- |
| **Psychiatric morbidity ^a^** | 304-305, 309, 313 | F10-F13, F15-F16, F19, F30-F34, F38-F43, F50, F53, F60, F90, O993, Z73, Z865 |  |  |
| **Involuntary childlessness** |  |  |  | Yes |
| **Vaginal delivery, vacuum extraction** |  | O814 |  |  |
| **Elective CS** |  | O820 |  | Yes |
| **Emergency CS** |  | O821 |  | Yes |
| **Gestational diabetes mellitus** | 648W | O244 |  |  |
| **Gestational hypertension or preeclampsia** | 642D-F | O10, O13-O15 |  |  |
| **Induction of delivery** |  | O61, O756B |  | Yes |
| **Dystocia** | 661A-B, 662C | O620-O622, O628-O629, O63 | DT037 | Yes |
| **Severe lacerations** | 664C, 665D-E | O702, O703, O713, O714 | MBB10, MBC00, MBC10, MBC33 |  |
| **Placental retention** | 667A | O73 | MBA00, MBA03, MBA10, MBA20, MBA30 |  |
| **Postpartum hemorrhage** | 666A-B | O72 |  |  |
| **Premature birth < 37 w** |  |  |  | Yes |
| **SGA** |  |  |  | Yes |
| **LGA** |  |  |  | Yes |
| **Apgar 5 minutes < 7** |  |  |  | Yes |
| **Composite worst-case variable ^b^** | 641C, 642F | O141, O142, O149, O15, O45, R568 |  | Yes |

*Note*. Cesarean section (CS), International Statistical Classification of Diseases and Related Health Problems (ICD), Medical Birth Register (MBR), small for gestational age (SGA), Swedish Classification of Health Interventions (KVÅ), large for gestational age (LGA). ^a^ Also including prescribed drugs of the Anatomical Therapeutic Chemical Classification (ATC) categories N05-N06. ^b^ Consisting of stillborn, eclampsia, severe PE, premature birth < 32 weeks, SGA below -2.5 SD (0.6%), placental abruption.
